# The secret lives of cancer cell lines

**DOI:** 10.1242/dmm.037366

**Published:** 2018-11-16

**Authors:** Robert E. Hynds, Elina Vladimirou, Sam. M. Janes

**Affiliations:** 1 CRUK Lung Cancer Centre of Excellence, UCL Cancer Institute, University College London, London WC1E 6JD, UK; 2 Cancer Evolution and Genome Instability Laboratory, The Francis Crick Institute, London NW1 1AT, UK; 3 Lungs for Living Research Centre, UCL Respiratory, University College London, London WC1E 6JF, UK

**Keywords:** Cancer, Cell line, Tissue culture, Tumor cell line, Genetic heterogeneity

## Abstract

The extent of genetic and epigenetic diversity between and within patient tumors is being mapped in ever more detail. It is clear that cancer is an evolutionary process in which tumor cell intrinsic and extrinsic forces shape clonal selection. The pre-clinical oncology pipeline uses model systems of human cancer – including mouse models, cell lines, patient-derived organoids and patient-derived xenografts – to study tumor biology and assess the efficacy of putative therapeutic agents. Model systems cannot completely replicate the environment of human tumors and, even within the same cancer model, data are often irreproducible between laboratories. One hypothesis is that ongoing evolutionary processes remain relevant in laboratory models, leading to divergence over time. In a recent edition of Nature, Ben-David and colleagues showed that different stocks of widely used cancer cell lines – a staple of cancer research over many decades – are highly heterogeneous in terms of their genetics, transcriptomics and responses to therapies. The authors find compelling evidence of positive selection based on ongoing mutational processes and chromosomal instability. Thus, the origin, culture conditions and cumulative number of population doublings of cell lines likely influence experimental outcomes. Here, we summarize the key findings of this important study and discuss the practical implications of this work for researchers using cell lines in the laboratory.

## Abstract (precis)

**Summary:** A recent study in Nature demonstrated that ongoing mutational processes cause significant heterogeneity of widely used cancer cell lines. In this Editorial, the authors discuss the implications of these findings for the cancer and cell biology fields.

To study human cancer biology, researchers can use patient biopsies or tissue from the surgical resection of tumors. However, this tissue represents a single snapshot in the life history of that tumor, typically at an advanced stage. In order to investigate the dynamic genetic and epigenetic course of cancer initiation, progression and metastasis, experimental model systems are required. More recent systems include genetically engineered mouse models (GEMMs) (Day et al., 2015), patient-derived xenografts (PDXs) (Byrne et al., 2017) and patient stem cell-derived organoids (Drost and Clevers, 2018), but by far the most commonly used remain cancer cell lines (van Staveren et al., 2009).

Cancer models are typically appraised in terms of how completely they recapitulate the features of the cancer of interest. Of course, none do so perfectly. GEMMs, for example, allow the monitoring of tumorigenesis in a longitudinal manner *in vivo*, but are limited by species differences in oncogenic pathogenesis, the shorter lifespan of mice and, often, by the artificial, simultaneous introduction of multiple oncogenic driver events. Cell lines have the advantage of being derived from patients and are more easily manipulated in the laboratory. Nevertheless, the cancer research community has long appreciated that cell lines do not completely recapitulate human disease (Hughes et al., 2007), as only a subset of patient tumors, and tumor cells, are amenable to growth on plastic tissue culture substrates. The establishment of pure, proliferative cultures of malignant cells leads to selection as the differentiated features of tumors are frequently lost, and we readily acknowledge that ‘substrains’ of cell lines exist, likely due to clonal evolution during long-term culture. Now, in experiments published in Nature (Ben-David et al., 2018), Uri Ben-David and colleagues have revealed the extent to which individual cancer cell lines are heterogeneous and continue to diversify during long-term cell culture (Fig. 1).

**Fig. 1.**
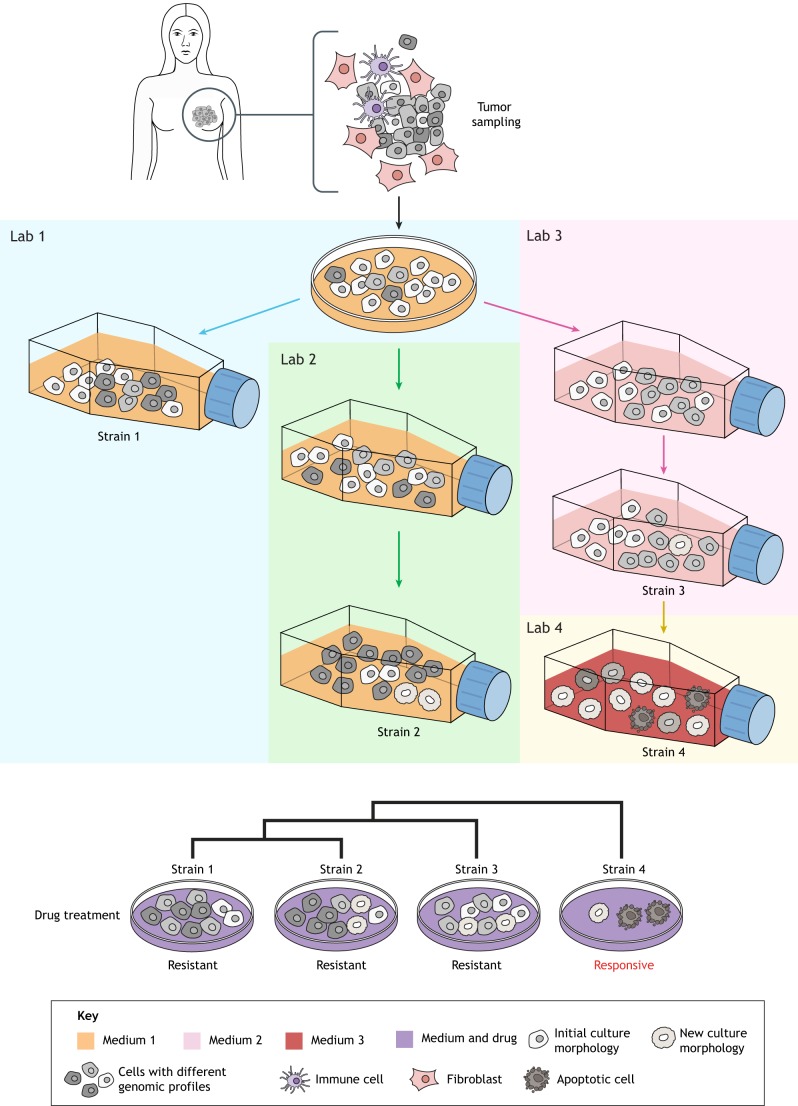
**Genomic diversity in cell line cultures.** Cell lines are proliferative cultures of transformed tumor cells derived from patient samples and grown on 2D tissue culture substrates. Selection occurs at multiple levels during cell line derivation: non-cancer cells are excluded and proliferative cells are selected for, leading to a bias for poorly differentiated cells. Ben-David and colleagues provide evidence that cell lines are genomically unstable during propagation with ongoing selection based on environmental factors, such as cell culture media types (Ben-David et al., 2018). This genomic heterogeneity, which arises through both mutational and chromosomal instability processes, alters the transcriptional profile and drug response of cell lines, and might explain how using the same cell lines in different studies can lead to contradictory results.

Initially, the authors tested the hypothesis of clonal variation within established cell lines by re-analyzing published whole-exome sequencing data, originating from the Broad and the Sanger Institutes' cancer cell line sequencing efforts, applying the same methodology to both data sets. Between the two sources of cell line data, a median of 19% of the detected non-silent mutations and 26% of gene copy number alterations (CNAs) were present in one data set but not the other. This discordance prompted the authors to examine the genetic variability within the cell lines in detail.

Laboratories around the world maintain different substrains of cell lines, so the authors sourced 27 independent vials of MCF7, an estrogen receptor-positive breast cancer cell line, predominantly from two major Broad Institute projects [Cancer Cell Line Encyclopedia (https://portals.broadinstitute.org/ccle) and Connectivity Map (https://clue.io/cmap)]. The MCF7 strains also demonstrated substantial heterogeneity in their mutational status, chromosomal rearrangements and CNAs. Deep, targeted sequencing of 447 genes revealed that just 35% of coding non-synonymous single-nucleotide variants (SNVs) and insertions and deletions were shared by all of the strains, while 29% were unique to a single substrain. Even when specific mutations were present in all strains, the allelic fraction, defined as the number of variant reads divided by the total number of reads at a specific genomic locus, varied substantially. The authors highlight an activating *PIK3CA* mutation (G1633A), for which the allelic fraction fluctuated between 0.21 and 0.7, as an example likely to have functional consequences. Differences in larger genomic rearrangements, such as arm-level copy number events, and differential copy number gains and/or losses in hundreds of genes were also evident among strains. Of those events, only 13% of gains and 21% of losses were present across all strains, with 7% of gains and 13% of losses being unique to a single strain. Importantly, these events affected genes such as *TP53*, *EGFR* and *PTEN*, which are known to be gained or lost during breast cancer pathogenesis. This indicated that individual existing strains of the MCF7 cell line might be more biologically relevant to the study of estrogen receptor-positive breast cancer than others.

Reassuringly, the overall genetic distance between substrains appeared to correlate with how recently the cell lines diverged from each other. This means that two strains growing in independent laboratories for 2 years likely have more in common than two strains that have been evolving in separate laboratories for 10 years. However, strains can diverge rapidly, as two versions of the cell line that were only a few passages apart had acquired an additional mutation in one of the genes included in the analysis. Such divergence could hinder the reproducibility of experiments and explain the discrepancies within experimental replicates.

Next, the authors demonstrated that positive selection, rather than stochastic variation, underlies the genetic heterogeneity of MCF7 strains. Barcoding experiments of five biological replicates grown in five different culture conditions demonstrated that the same pre-existing subclones were being selected for in each culture condition. These data indicate that even minor changes in cell culture protocols can alter the cell line genotype through selection. Furthermore, genetic heterogeneity is re-established when culturing single cell-derived clones over time. This, combined with the observation that time in culture, rather than freeze-thaw cycles, was the main contributor to genetic divergence between strains, supports the authors' conclusion that ongoing genomic instability, rather than stochastic bottlenecks imposed by culture routines, underlies the variation in subclonal composition of MCF7 cells.

Beyond the measured genomic variation, and despite identical culture conditions, the 27 MCF7 strains also exhibited transcriptomic variation, albeit with an overall similarity in global gene expression profiles. Clustering by transcriptomic profiles was very similar to clustering by genomic distance, suggesting that differential pathway activation due to varied genetic background underlies the gene expression differences between strains. However, variations in cell culture substrates, media and culture environments likely mean that this study, performed in a single laboratory using identical culture conditions, underestimated the inter-laboratory variability in gene expression of any given cell line. Using quantitative live-cell imaging, the authors measured several properties linked to cellular function. Consistent with anecdotal experiences of working with cell lines, the authors found that the doubling times and morphology of the MCF7 cells were widely variable among strains, and also correlated with the observed genomic and gene expression variation.

Ben-David et al. validated their MCF7 findings in the *KRAS*-mutant lung adenocarcinoma cell line A549 (Giard et al., 1973), which is widely used in lung cancer research. They observed a similar variability between 23 A549 strains; the key lung adenocarcinoma driver gene *CDKN2A* was lost in five of the strains and KRAS pathway components were highly variable in transcriptomic analyses. Targeted sequencing results from 11 further cell lines, and similar results from an investigation of the genomic, transcriptomic and phenotypic heterogeneity of HeLa cells from 13 independent laboratories (Liu et al., 2018), suggest that Ben-David et al.’s findings have universal relevance to cell line culture. Assessing the extent and importance of this variability for specific cell lines will be a new challenge for the field.

Of particular concern for cell biologists, the observed heterogeneity was not limited to transformed cells: 15 strains of MCF10A, a spontaneously immortalized ‘normal’ human mammary cell line, also demonstrated genomic heterogeneity levels comparable to those of transformed cell lines. Another recent study found that primary murine epithelial cells were more prone to chromosome mis-segregation in 2D cultures than when grown in 3D organoid culture or *in vivo*, where tissue architecture maintains a high chromosome segregation fidelity through the enhanced correction of kinetochore merotelic attachments (Knouse et al., 2018). In this sense, the artificial nature of 2D cell culture on plastic might recapitulate an important aspect of epithelial carcinogenesis – the lack of epithelial polarity and organized tissue structure. Some primary cell cultures, such as mouse embryonic fibroblasts (Berenjeno et al., 2017), display high rates of chromosomal instability. Clinically, however, the success of primary epithelial stem cell therapies in patients (Hynds et al., 2018) – where engrafted cells are karyotypically normal – suggests that this might not be a universal 2D cell culture phenomenon. Tissue- or species-specific differences may affect chromosomal stability profiles *in vitro*, and optimized 2D culture conditions might reduce the frequency of mis-segregation events. Moreover, the observation that unstable, late-stage tumors give rise to more unstable cell lines (Ben-David et al., 2018), coupled with previous data showing that tetraploid genomes undergo more rapid copy number evolution than diploid genomes within the HCT-116 cell line (Dewhurst et al., 2014), suggests that the intrinsic genomic (in)stability of the initiating population is relevant to the future evolution of a cell line.

Cell lines are an important source of information when investigating potential new therapies, so a crucial question arising from Ben-David et al.’s initial experiments is whether the genomic and transcriptomic heterogeneity of cell lines affects our ability to appraise new therapeutic agents. In a screen of 321 compounds, the drug response of the different MCF7 strains was highly variable: among the 55 compounds that inhibited the growth of at least one strain by more than 50%, 48 of these showed <20% growth inhibition in at least one other strain. These differences in drug response are underpinned by genetic and transcriptomic differences and go some way to explaining the frequent lack of reproducibility between laboratories. The results also have implications for studies of drug resistance, as previous studies showed that rare resistant clones are present within cell lines and emerge to become dominant under heavy selection, such as with cancer cells harboring activating mutations in *EGFR* grown under EGFR inhibition (Hata et al., 2016). If different subclonal compositions exist in different laboratories, then conflicting outcomes, even in response to the same therapy, are likely.

The formal recognition of the heterogeneity propagated by cell line culture adds to our uncertainty about how to interpret data from cancer model systems, particularly in light of the recent advances unveiling the extent of intratumoral heterogeneity detected in patient tumors. The problems with cell culture systems highlighted by Ben-David et al. strongly support efforts to systematically develop new patient-derived cancer models to reduce our reliance on poorly defined cell lines that were established before the emergence of next generation sequencing and have been subjected to extensive ongoing selection. These new ‘close-to-patient’ models likely reflect the patient's disease more accurately (Hidalgo et al., 2014; Weeber et al., 2015), even though they are probably subject to the same evolutionary pressures as conventional cancer cell lines during propagation. Although PDX models are broadly thought to preserve the genotype of the tumor from which they are derived (Hidalgo et al., 2014), there is some evidence that, similar to metastases in patients, they might arise from a subclone of the primary tumor (Ding et al., 2010). Characterizing greater numbers of patients in detail will clarify this issue and shed light on the possible differences in selection pressures between subcutaneous and orthotopic PDX models (Stewart et al., 2017). Ben-David et al. have previously demonstrated that ongoing, *ex vivo* evolution is relevant in PDX models, where CNAs that were not found in the original tumor emerged after serial passaging (Ben-David et al., 2017). Detailed and repeated characterization of new cell and organoid lines is also important for reproducibility, as contamination and overgrowth of cancer cell cultures with tumor-associated fibroblasts or normal epithelial cells occurs frequently (Gao et al., 2017; Sachs et al., 2018).

We believe that these data signal an end to the era in which researchers informally acknowledge that different strains of cancer cell lines are heterogeneous and unstable over long-term passage, but in practice treat them as though they were clonal entities. There are already established measures to minimize irreproducibility between laboratories, such as cell line identification (Fusenig et al., 2017), mycoplasma testing (Lorsch et al., 2014), the use of carefully validated reagents (Shah et al., 2018) and robust statistical standards (Loken and Gelman, 2017; Weissgerber et al., 2017). In addition to these, researchers can take several practical steps to mitigate and, perhaps, also utilize the heterogeneity of cell line strains. When best practice guidelines (Geraghty et al., 2014) are re-written, they might now recommend the following:

(1) For existing cell lines, each laboratory's strain should be profiled. Reference genomes for cell lines [e.g. the Broad Institute's Cancer Cell Line Encyclopedia (https://portals.broadinstitute.org/ccle) or the Sanger Institute's Cell Lines Project (https://cancer.sanger.ac.uk/cell_lines)] should be used for broad direction but specific mutation/pathway status should be confirmed locally.
(2) On adoption of new cell lines, identical cryovials of very early passage cell lines should be established on arrival and later passage working stocks derived from just one of these. Master stocks could then be used for validation of key findings prior to publication.
(3) More rigorous monitoring and reporting of population doublings should become the norm, and substitute the current ‘passage number’ convention, which is imprecise. Cell lines should preferably be used within a defined window to minimize genetic drift.
(4) The optimization, agreement and implementation of international ‘standard’ culture conditions for individual cell lines together with the documentation of heterogeneity metrics (e.g. measures of genomic divergence per passage in those conditions) in datasheets.
(5) The reassessment of genomic landscape after significant culture bottlenecks induced by selection and/or single cell cloning (e.g. in CRISPR-Cas9 gene editing experiments).

In summary, Ben-David et al. reveal the scale of heterogeneity between strains of cancer cell lines and the ongoing nature of the processes that underlie this heterogeneity. Importantly, these processes are unlikely to be similar to the ongoing genomic processes in human tumors, and mean that as cell lines – already an idiosyncratic representation of a single patient's cancer – are expanded long-term, they become even less representative of human disease. Importantly, recognizing the variability within laboratory cancer models, whether *in vitro* or *in vivo*, presents opportunities for researchers to improve the reporting and reproducibility of pre-clinical cancer research.
